# Identification of quantitative trait loci for body temperature, body weight, breast yield, and digestibility in an advanced intercross line of chickens under heat stress

**DOI:** 10.1186/s12711-015-0176-7

**Published:** 2015-12-17

**Authors:** Angelica Van Goor, Kevin J. Bolek, Chris M. Ashwell, Mike E. Persia, Max F. Rothschild, Carl J. Schmidt, Susan J. Lamont

**Affiliations:** Department of Animal Science, Iowa State University, Ames, IA USA; Department of Animal Science, University of California, Davis, CA USA; Department Poultry Science, North Carolina State University, Raleigh, NC USA; Department of Animal and Poultry Sciences, Virginia Polytechnic Institute and State University, Blacksburg, VA USA; Department of Animal and Food Sciences, University of Delaware, Newark, DE USA

## Abstract

**Background:**

Losses in poultry production due to heat stress have considerable negative economic consequences. Previous studies in poultry have elucidated a genetic influence on response to heat. Using a unique chicken genetic resource, we identified genomic regions associated with body temperature (BT), body weight (BW), breast yield, and digestibility measured during heat stress. Identifying genes associated with a favorable response during high ambient temperature can facilitate genetic selection of heat-resilient chickens.

**Methods:**

Generations F18 and F19 of a broiler (heat-susceptible) × Fayoumi (heat-resistant) advanced intercross line (AIL) were used to fine-map quantitative trait loci (QTL). Six hundred and thirty-one birds were exposed to daily heat cycles from 22 to 28 days of age, and phenotypes were measured before heat treatment, on the 1st day and after 1 week of heat treatment. BT was measured at these three phases and BW at pre-heat treatment and after 1 week of heat treatment. Breast muscle yield was calculated as the percentage of BW at day 28. Ileal feed digestibility was assayed from digesta collected from the ileum at day 28. Four hundred and sixty-eight AIL were genotyped using the 600 K Affymetrix chicken SNP (single nucleotide polymorphism) array. Trait heritabilities were estimated using an animal model. A genome-wide association study (GWAS) for these traits and changes in BT and BW was conducted using Bayesian analyses. Candidate genes were identified within 200-kb regions around SNPs with significant association signals.

**Results:**

Heritabilities were low to moderate (0.03 to 0.35). We identified QTL for BT on *Gallus gallus* chromosome (GGA)14, 15, 26, and 27; BW on GGA1 to 8, 10, 14, and 21; dry matter digestibility on GGA19, 20 and 21; and QTL of very large effect for breast muscle yield on GGA1, 15, and 22 with a single 1-Mb window on GGA1 explaining more than 15 % of the genetic variation.

**Conclusions:**

This is the first study to estimate heritabilities and perform GWAS using this AIL for traits measured during heat stress. Significant QTL as well as low to moderate heritabilities were found for each trait, and these QTL may facilitate selection for improved animal performance in hot climatic conditions.

## Background

The climate is becoming increasingly warmer, according to the Intergovernmental Panel on Climate Change, and the global average temperature will continue to increase by 0.2 °C per decade. Heat stress in poultry impacts animal production and welfare and, in the poultry industry, it causes an estimated economic loss of $125 to 165 million in the US, with the broiler industry alone accounting for $58.1 million [1]. In 2007, an extreme heat wave in California resulted in more than 700,000 deaths in poultry [2] and in 2009, over 1.5 million layer hens died during a summer heat wave (National Oceanic and Atmospheric Association).

Production losses due to heat stress may result from mortality, reduced body weight, reduced egg production, reduced feed intake, and higher feed to gain ratio. A recent study on broilers that were exposed to chronic heat stress from 1 to 42 days of age showed a reduced body weight (32.6 %), increased feed conversion ratio (25.6 %), and reduced feed intake (16.4 %) [3], and another study using shorter periods of heat stress on younger birds, from 2 to 4 weeks of age showed a reduced feed intake by 14 % [4]. In a paired feed study, genetically lean broilers that were exposed to chronic heat stress, from hatch to 9 weeks of age showed increased weight gain and feed efficiency compared to less lean counterparts, which supports the hypothesis that increased fat accretion is inversely related to thermo-regulation [5]. Previous studies in poultry have elucidated a genetic influence on response to heat. Layers that were divergently selected for heat tolerance displayed differences in survivability during increased heat conditions [6]. Significant differences in production traits have been found between a commercially fast growing chicken line and a local chicken breed from China during heat stress [7]. Microsatellites were used to identify quantitative trait loci (QTL) for several traits measured during heat stress in a Japanese quail F2 intercross including body weight (BW), feed intake, and body temperature (BT) [8]. Because in poultry, response to heat stress involves a genetic component, it is possible to use genomic selection for heat tolerance, which will increase accuracies and response to selection [9]. To increase our understanding of the genetic influence on response to heat stress in chickens, we used the F18 and F19 generations of a broiler (heat-susceptible) × Fayoumi (heat-resistant) advanced intercross line (AIL) and an environmentally-controlled experiment to identify genomic markers related to response to high ambient temperatures.

Chickens of this AIL were exposed to high ambient temperatures for 7 days during which BW, BT, breast yield, and digestibility were measured. These traits, as well as the changes in BT and BW due to heat treatment, were used for genome-wide association studies (GWAS). The genes and markers associated with thermal tolerance can help elucidate the genetic architecture of traits involved in heat stress and, subsequently, be used to breed more heat-resilient chickens.

## Methods

### Chicken lines

All animal experiments were approved by the Institutional Animal Care and Use Committee at Iowa State University: Log #4-11-7128-G. We used the F18 and F19 generations of an AIL between two highly divergent chicken lines for thermo-tolerance, i.e. it was created by crossing a single broiler sire to six highly inbred Fayoumi dams [10]. Although this population has limited variability due to the initial mating, the broiler sire was characterized by the offsprings’ phenotypic means and variances of body composition phenotypes, which showed that it was representative of the entire broiler population [10]. We hypothesize that the highly inbred Fayoumi breed became fixed for alleles that had the highest frequency in the founder line. Thus, this population is a powerful resource to identify QTL. The broiler breed has been commercially selected for muscle accretion, whereas the Fayoumi breed has not undergone commercial selection. The Fayoumi breed originated in the Fayoum region of Egypt that is characterized by a high-temperature climate and thus this breed has undergone natural selection for tolerance to heat. Birds were reared on floor pens with wood shavings and had ad libitum access to water and corn-soy feed that met all NRC requirements for the duration of the study [11].

### Heat stress experimental design

We used birds from two generations with each generation producing two hatches. Six hundred and thirty-one birds from the four hatches were used for independent heat stress experiments (four replicates). Six hundred animals from 17 sire families were phenotyped for BT, BW, and breast yield. Digestibility measurements were available for 461 animals from 14 sire families. At 17 days of age, birds were transferred to environmentally-controlled chambers and acclimated for 5 days. There were four chambers, each containing six pens, per replicate. From days 22 to 28 of age, the chambers were heated to 35 °C for 7 h per day and remained at 25 °C at all other times.

### Phenotypic measurements

Cloacal BT was measured by inserting a digital thermometer approximately 2.5 cm into the cloaca on days 20, 22, and 28 of age. The precision of the digital thermometer was 0.1 °C. BW was measured using a digital scale on days 21 and 28 of age. Breast yield (%) was determined by weighing one half of the pectoralis major and minor muscle, multiplying this value by two, and then dividing it by the total BW on day 28. Dry matter digestibility was measured as described in [12]. Briefly, dry matter was determined by drying ileal and feed samples for 24 h at 110 °C. Titanium dioxide was used as a marker for both ileal and feed samples and was analysed as described in [13]. Dry matter digestibility was calculated by the following equation [12]:

$$\left [ {\frac{{{\rm \%\, Diet \,DM} - ( {\frac{{\rm \%\, Fecal \,DM \; \times \% \,Diet \,Ti}}{{\rm \% \,Fecal \,Ti}}} )}}{{\rm \% \,Diet \,DM}}}\right ]\; \times \;100,$$

where DM is dry matter and Ti is titanium dioxide. Dry matter digestibility was log-transformed to obtain a normal distribution of the data and the transformed data was used for all downstream analyses.

### DNA isolation and genotyping

Blood was collected from the wing vein by using an EDTA-coated syringe and needle, and then stored at −20 °C. DNA was extracted using a salting out method. Briefly, whole blood was incubated with lysis buffer containing proteinase K. Proteins were precipitated out using 5 M NaCl and the supernatant was recovered. 70 % ethanol was added to the supernatant to precipitate DNA. DNA isolated from 468 AIL, six broiler, and six Fayoumi chickens was genotyped using the Affymetrix 600 K chicken SNP (single nucleotide polymorphism) axiom array [14] by GeneSeek Inc., Lincoln, NE. SNP chromosomal locations were based on the Gallus_gallus_4.0 assembly through Ensembl.

### Statistical analyses

Means, standard errors, fixed effects, and covariates for the GWAS analyses were calculated based on ANOVA (analysis of variance) estimates, and significant terms were included as fixed effects with a P value less than 0.05 using JMP statistical software [15]. Heritability was estimated using a single-trait animal model in ASReml [16]. For all phenotypes, sex was fitted as a fixed effect, while chamber nested within replicate and within animal were fitted as random effects. To estimate BW21 heritability, dam was fitted as a random effect. For the GWAS for BT, the closest BW measurement in days was fitted as a covariate.

Genotyping console (Affymetrix) software was used to obtain genotyping calls and to perform quality control based on whole animal DishQC score ≥0.7. The SNPolisher (Affymetrix) R package was used to perform quality control of individual SNPs for all the animals that passed the DishQC criterion. For SNP genotypes to be included in the analysis, SNP call rate had to be greater than 95 % and minor allele frequency (MAF) higher than 5 %.

GWAS for phenotypic traits with SNP genotypes was done using GenSel software [17]. Bayes B, which fits all SNPs simultaneously as random effects, was used for the analysis. The following mixed model was used for the GWAS:

$${\mathbf{y = Xb}}{ + }\sum\limits_{\text{j}}^{\text{k}} {{\mathbf{z}}_{\text{j}} \upalpha_{\text{j}} \delta_{\text{j}} { + } {\upvarepsilon} },$$

where **y** is a vector of phenotypes, **X** is an incidence matrix to account for fixed effects on phenotypes, **b** is a vector of fixed effects, **z**_j_ is a vector of genotypes for SNP j based on the number of B alleles (−10, 0, +10, or the average of the genotypes at SNP j), α_j_ is the allele substitution effect for SNP j, δ_j_ is a parameter that indicates whether SNP j was included in the Markov chain Monte Carlo (MCMC) chain, and ε is the error associated with the analysis. For one analysis per trait, a total of 41,000 MCMC iterations were completed and the first 1000 iterations were discarded.

SNPs were split into 1001 non-overlapping 1-Mb windows across the genome. Thus, the windows that have the SNP, which is most frequently included in the MCMC iterations (post-burn-in), are predicted to have an effect on the phenotype. δ_j_ was set so that π = 0.9978 to avoid fitting more SNPs than the number of animals in a given iteration. Using a true infinitesimal model, each window is expected to explain 0.1 % (100 %/1001) of the genetic variation; therefore, a 1-Mb window was considered significant if it explained more than 0.5 % of the total genetic variation.

### Candidate genes

For each trait, the window explaining the largest percentage of genetic variation was investigated, and, within this window, the SNP that was most frequently included in the model was identified. Then, all annotated genes within 200 kb (100 kb upstream and 100 kb downstream) of that SNP were identified using ENSEMBL biomart [18]. We chose a 200-kb window based on the average linkage disequilibrium (LD) in commercial broiler populations i.e., less than 1 cM on average [19], and on the fact that the chicken genome contains 250 kb per cM on average [20]. In the F18 and F19 AIL chickens, LD was expected to cover a shorter distance than in the commercial broiler population because of their unique population structure and the large number of generations in which recombination occurred.

## Results

### Phenotypic measurements and heritabilities

Phenotypic means, standard errors, ranges, and heritabilities are in Table 1. BT measurements had low heritabilities that ranged from 0.03 to 0.11. Changes in BT after acute heat (1 day) and chronic heat (7 days) treatments had low heritabilities of 0.03 with large standard errors and were not statistically different from 0. BW measurements had moderate heritabilities that ranged from 0.15 to 0.35. Breast yield and dry matter digestibility, both measured on day 28, had moderate heritabilities of 0.15 and 0.33, respectively.

**Table 1:** Phenotypic means and heritabilities (h^2^)

Trait	Mean ± SEM (range)	h^2^ (SE)
BT20	42.3 ± 0.01 (41.5–42.9)	0.11 (0.06)
BT22	42.4 ± 0.02 (41.2–43.2)	0.10 (0.06)
BT22-20	0.1 ± 0.02 (−1.4 to 1.5)	0.03 (0.04)
BT28	42.3 ± 0.01 (41.4–43.1)	0.10 (0.06)
BT28-20	−0.02 ± 0.02 (−1.1 to 1.3)	0.03 (0.04)
BW21	253.6 ± 1.58 (157.6–352.0)	0.24 (0.17)
BW28	402.6 ± 2.58 (238.7–555.3)	0.35 (0.11)
BW28-21	149.2 ± 1.57 (48.1–203.3)	0.15 (0.11)
Digestibility	90.6 ± 0.98 (86.2–95.8)	0.33 (0.14)
Percent breast weight	8.81 ± 0.03 (5.1–12.6)	0.15 (0.08)

Body temperature (BT) measured on days 20, 22, 28, and the differential 28-20; body weight (BW) measured on days 21, 28, and the differential 28-21; digestibility, measured from ileal content, on day 28; percent of breast weight, calculated from percent of total body weight, and measured on day 28

### Genotypes

Of the 480 birds that were genotyped, 458 AIL and all 12 parental line birds passed the whole animal DishQC criterion. Of the 580,961 SNPs on the array, filtering based on a SNP call rate greater than 95 % removed a small proportion, i.e. 59,789 SNPs, while filtering based on MAF removed a much larger proportion, i.e. 311,055 SNPs, thus 210,117 SNPs remained for subsequent analyses.

### GWAS

The detailed results for each window that explained a significant percentage of the genetic variation (>0.5 %) and the SNP within each window that had the highest effect on the trait are in Table 2. To increase clarity, significant consecutive windows were designated as a single QTL. In total, 35 QTL were identified across all traits and measurement phases.

**Table 2:** Identified windows that explain a significant percentage (≥0.5 %) of the genetic variance

Windows explaining ≥0.5 % of genetic variance						SNP with highest model freq within window			
Trait^a^	Chr	Pos (Mb)	% of genetic variance explained	Nb of SNPs	Freq of iterations with (P > 0)^b^	SNP name^c^	SNP pos (bp)^d^	Model freq^e^	Allele freq^f^
BT20	1	70	0.66	154	0.37	AX-75508759	70481838	0.0037	0.684
BT20	1	77	0.57	147	0.34	AX-75522132	77489841	0.0035	0.128
BT20	14	3	0.68	444	0.67	AX-75795199	3948035	0.0034	0.624
BT20	14	4	1.02	387	0.63	AX-75797528	4705324	0.0044	0.254
BT22	15	8	0.72	232	0.44	AX-75845885	8214511	0.0038	0.489
BT28	15	8	1.01	232	0.55	AX-80869149	8162150	0.0044	0.513
BT28	15	9	0.94	250	0.54	AX-80984099	9043951	0.0035	0.492
BT28	26	1	0.62	345	0.58	AX-76333878	1820964	0.0068	0.719
BT28-20	14	3	0.51	444	0.63	AX-75792785	3211698	0.0036	0.383
BT28-20	27	2	0.58	650	0.76	AX-76359339	2735538	0.0032	0.477
BW21	1	130	0.66	186	0.50	AX-75254200	130612102	0.0428	0.459
BW21	2	45	0.77	198	0.47	AX-76097146	45668714	0.0091	0.391
BW21	2	46	2.37	173	0.61	AX-76098569	46452516	0.0146	0.408
BW21	2	47	0.65	203	0.46	AX-76099514	47012242	0.0084	0.407
BW21	4	29	0.75	126	0.42	AX-76640215	29660145	0.0083	0.569
BW21	6	17	0.58	191	0.48	AX-76911192	17573301	0.0041	0.37
BW21	6	18	0.57	216	0.53	AX-76914599	18873299	0.0053	0.381
BW21	6	19	0.55	184	0.45	AX-76916544	19707190	0.0052	0.438
BW21	6	20	1.27	319	0.69	AX-76918160	20296520	0.0048	0.619
BW21	7	25	0.62	220	0.51	AX-77014466	25790174	0.0054	0.543
BW21	8	19	0.53	224	0.52	AX-77082473	19967735	0.0061	0.475
BW21	8	20	0.59	324	0.55	AX-77082837	20103726	0.0081	0.567
BW21	14	2	0.5	245	0.55	AX-75789727	2184924	0.0039	0.752
BW28	1	129	0.69	216	0.56	AX-75251743	129532260	0.0070	0.497
BW28	1	130	0.5	186	0.47	AX-80745974	130704373	0.0052	0.542
BW28	1	175	0.51	218	0.53	AX-75350071	175304877	0.0040	0.501
BW28	2	46	0.57	173	0.48	AX-76098569	46452516	0.0096	0.408
BW28	3	7	0.56	269	0.55	AX-76560040	7975734	0.0102	0.472
BW28	4	35	0.8	278	0.59	AX-80752029	35879320	0.0057	0.207
BW28	4	36	1.05	292	0.63	AX-80949517	36028465	0.0060	0.790
BW28	5	2	0.65	154	0.39	AX-76800406	2270651	0.0051	0.479
BW28	5	4	0.7	234	0.54	AX-76843401	4221327	0.0049	0.539
BW28	6	17	1.1	191	0.52	AX-80910640	17891764	0.0050	0.377
BW28	6	18	1.67	216	0.59	AX-76914769	18937498	0.0075	0.371
BW28	6	19	2.48	184	0.59	AX-76915818	19346379	0.0081	0.624
BW28	6	20	2.11	319	0.72	AX-76918815	20548526	0.0053	0.404
BW28	6	21	0.77	173	0.44	AX-76921099	21508397	0.0053	0.405
BW28	26	3	0.54	616	0.78	AX-76340801	3344288	0.0045	0.404
BW28-21	1	2	1	185	0.47	AX-75406964	2188529	0.0047	0.394
BW28-21	4	35	1.2	278	0.57	AX-76651146	35184184	0.0052	0.335
BW28-21	4	36	1.36	292	0.62	AX-80788958	36098921	0.0050	0.715
BW28-21	6	17	0.62	191	0.43	AX-76911330	17642627	0.0038	0.622
BW28-21	6	18	0.86	216	0.49	AX-80836439	18927710	0.0040	0.368
BW28-21	6	19	1.31	184	0.47	AX-76915818	19346379	0.0056	0.624
BW28-21	6	20	1.29	319	0.63	AX-76919341	20762964	0.0037	0.595
BW28-21	6	21	0.55	173	0.40	AX-76921368	21613870	0.0038	0.598
BW28-21	10	4	0.56	548	0.77	AX-75184920	4022205	0.0038	0.258
BW28-21	21	1	0.53	658	0.78	AX-80870523	1549522	0.0034	0.338
BW28-21	21	5	0.64	656	0.76	AX-76253089	5768734	0.0034	0.298
Digestibility	19	9	0.63	207	0.41	AX-75948077	9760405	0.0035	0.510
Digestibility	20	13	0.62	367	0.61	AX-76206206	13442859	0.0034	0.446
Digestibility	21	5	0.53	656	0.79	AX-76253122	5775142	0.0032	0.327
% Breast weight	1	42	0.58	107	0.27	AX-75450269	42731066	0.0035	0.350
% Breast weight	1	43	0.97	116	0.30	AX-75452817	43915634	0.0046	0.629
% Breast weight	1	44	15.35	158	0.74	AX-75454603	44752476	0.0220	0.428
% Breast weight	1	45	5.73	177	0.50	AX-75455154	45032476	0.0133	0.429
% Breast weight	1	46	1.85	176	0.46	AX-75457992	46437656	0.0056	0.401
% Breast weight	15	10	0.56	400	0.67	AX-75815669	10006485	0.0040	0.833
% Breast weight	22	3	1.11	573	0.79	AX-76267275	3079947	0.0052	0.542

^a^Body temperature (BT) measured on days 20, 22, 28, and the differential 28-20; body weight (BW) measured on days 21, 28, and the differential 28-21; digestibility, measured from ileal content, on day 28; percent of breast weight, calculated from percent of total body weight, and measured on day 28

^b^Frequency with which the window was included in the MCMC iterations (post-burn-in)

^c^SNP within the specified window which was most frequently included in the MCMC iterations (post-burn-in), and is therefore predicted to have an effect on the phenotype

^d^Position of SNPs in base pairs on *Gallus gallus* (version 4.0) chromosomes

^e^Frequency with which the SNP was included in the MCMC iterations (post-burn-in)

^f^Allele frequency of the SNP within the genotyped population (N = 458)

**Fig. 1:**
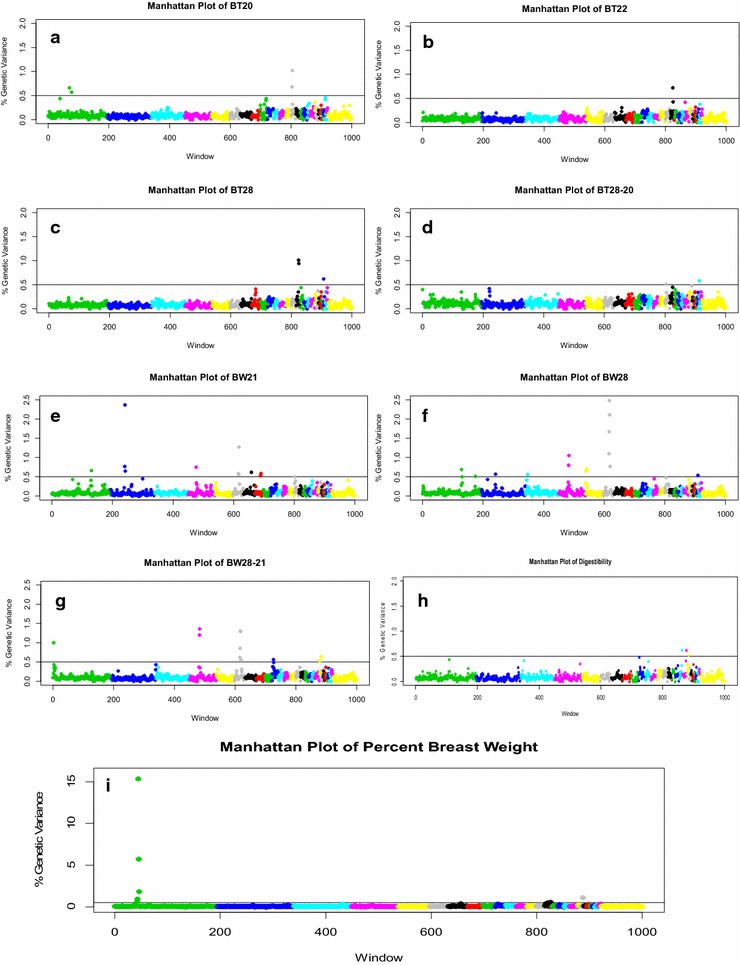
Genome-wide plot of percentage of genetic variance for traits measured during heat stress. Traits were measured before heat treatment (day 20 or 21), during acute heat treatment (day 22) and chronic heat treatment (day 28), and the differentials between trait measurements due to 1 day of heat treatment (day 22–20) and to 7 days of heat treatment (day 28–20 or 28–21) were calculated. Only traits that reached significance in the GWAS (≥0.05 % of the genetic variation) are displayed. Plots for body temperature (BT) measured on days 20, 22, 28, and the differential 28-20 (**a**–**d**); plots for body weight (BW) measured on days 21, 28, and the differential 28–21 (**e**–**g**); plot for digestibility, measured from ileal content, on day 28 (H); plot for  % of breast weight (**i**), calculated from  % of total body weight, and measured on day 28. Results show the percentage of genetic variance that is explained by each non-overlapping 1-Mb window, labelled by the index number of the windows coloured and ordered by chromosome (1–27, and Z)

Eight QTL were identified for BT phenotypes (Fig. 1a–d), i.e. (1) four for BT20 with two on GGA1 (GGA for *Gallus gallus* chromosome), one on GGA14, and one on GGA15; (2) two for BT28 with one on GGA15 and one on GGA26; and (3) two for BT28-20 (change in BT measured at pre-heat treatment and after 1 week of heat treatment) with one on GGA14 and one on GGA27. No QTL was found for BT22-20 (change in BT measured at pre-heat treatment and after 1 day of heat treatment). QTL co-localizations were identified on GGA14 for BT20 and BT28-20 and on GGA15 for BT20 and BT28.

Twenty-one QTL for BW phenotypes were identified, i.e. (1) seven for BW21 with one each on GGA1, 2, 4, 6, 7, 8, and 14; (2) nine for BW28 with two on GGA1, two on GGA5, and one each on GGA2, 3, 4, 6, and 26; and (3) five for BW28-21 (change in BW measured at pre-heat treatment and after 1 week of heat treatment) with one each on GGA1, 4, 6, 10, and 21 (Fig. 1e–g). QTL for all BW phenotypes co-localized on GGA6 in a region containing four adjacent 1-Mb windows. QTL also co-localized on GGA1 for BW21 and BW28 and on GGA4 for BW28 and BW28-21.

Three QTL were identified for digestibility with one each on GGA19, 20 and 21 (Fig. 1h). QTL co-localized for digestibility and BW28-21 on GGA21.

For breast yield, an economically important trait, three QTL were identified with one each on GGA1, 15, and 22 (Fig. 1i). The QTL on GGA15 included five adjacent 1-Mb windows and cumulatively accounted for 24.5 % of the genetic variation. The most significant single 1-Mb window in this region accounted for 15.4 % of the genetic variation. QTL co-localization was not identified between breast yield and any of the other traits measured in the current study.

### Candidate genes

For each trait, positional candidate genes were identified within a 200 kb region i.e. 100 kb upstream and 100 kb downstream of the SNP with the highest effect within the 1-Mb window that explained the highest percentage of genetic variation. Fifty annotated genes were identified (see Table 3). For BW measurements, five, four, and one positional candidate genes for BW21, BW28, and BW28-21, respectively, were found among the 18, 24, and six genes that were located within the corresponding 1-Mb windows. For BT measurements, seven, seven and 11 positional candidate genes for BT20, BT28, and BT28-20, respectively, were found among the 48, 38, and 29 genes that were located within the corresponding 1-Mb windows. For dry matter digestibility and breast yield, six and four positional candidate genes, respectively, were identified among the 19 and 10 genes that were located within the corresponding 1-Mb windows.

**Table 3:** Positional candidate genes categorized by function for windows explaining the largest percentage of genetic variation

Function	Trait	Gene name	Description
Disruption of DNA synthesis, transcription, RNA processing, and translation	BW21	HHEX	Gallus gallus hematopoietically expressed homeobox (HHEX), mRNA. [Source:RefSeq mRNA;Acc:NM_205252]
	BT20	MED9	Gallus gallus mediator complex subunit 9 (MED9), mRNA. [Source:RefSeq mRNA;Acc:NM_001277637]
	BT22	MED15	Mediator complex subunit 15 [Source:HGNC Symbol;Acc:HGNC:14248]
	BT28-20	RNF113A	Gallus gallus ring finger protein 113A (RNF113A), mRNA. [Source:RefSeq mRNA;Acc:NM_001004396]
	BT28-20	DDX42	Gallus gallus DEAD (Asp-Glu-Ala-Asp) box polypeptide 42 (DDX42), mRNA. [Source:RefSeq mRNA;Acc:NM_001030926]
	Digestibility	MED31	Mediator complex subunit 31 [Source:HGNC Symbol;Acc:HGNC:24260]
	Breast yield	MRPL42	Mitochondrial ribosomal protein L42 [Source:HGNC Symbol;Acc:HGNC:14493]
Disruption of progression through the cell cycle	BT22 & BT28	TBX6	Gallus gallus T-box 6 (TBX6), mRNA. [Source:RefSeq mRNA;Acc:NM_001030367]
	BT22	KLHL22	Kelch-like family member 22 [Source:HGNC Symbol;Acc:HGNC:25888]
	BT28-20	LIMD2	Gallus gallus LIM domain containing 2 (LIMD2), mRNA. [Source:RefSeq mRNA;Acc:NM_001006330]
	BT28-20	STRADA	Gallus gallus STE20-related kinase adaptor alpha (STRADA), mRNA. [Source:RefSeq mRNA;Acc:NM_001012844]
	Digestibility	KIAA0753	KIAA0753 [Source:HGNC Symbol;Acc:HGNC:29110]
Increase protein degradation by ubiquitination	BW21	MARCH5	Gallus gallus membrane-associated ring finger (C3HC4) 5 (MARCH5), mRNA. [Source:RefSeq mRNA;Acc:NM_001012906]
	BW28	PCGF5	Gallus gallus polycomb group ring finger 5 (PCGF5), mRNA. [Source:RefSeq mRNA;Acc:NM_001277361]
	BW28	HECTD2	HECT domain containing E3 ubiquitin protein ligase 2 [Source:HGNC Symbol;Acc:HGNC:26736]
	BT20	USP22	Ubiquitin carboxyl-terminal hydrolase [Source:UniProtKB/TrEMBL;Acc:F1NG36]
Membrane permeability and ions	BW28-21	GRID2	Glutamate receptor, ionotropic, delta 2 [Source:HGNC Symbol;Acc:HGNC:4576]
	BT20	PEMT	Gallus gallus phosphatidylethanolamine N-methyltransferase (PEMT), nuclear gene encoding mitochondrial protein, mRNA. [Source:RefSeq mRNA;Acc:NM_001006164]
	BT28-20	CYB561	Cytochrome b561 [Source:HGNC Symbol;Acc:HGNC:2571]
	BT28-20	KCNH6	Potassium voltage-gated channel, subfamily H (eag-related), member 6 [Source:HGNC Symbol;Acc:HGNC:18862]
	BT28-20	CCDC47	Coiled-coil domain containing 47 [Source:HGNC Symbol;Acc:HGNC:24856]
	BT28-20	MYL4	Myosin, light chain 4, alkali; atrial, embryonic [Source:HGNC Symbol;Acc:HGNC:7585]
	Digestibility	SLC13A5	Solute carrier family 13 (sodium-dependent citrate transporter), member 5 [Source:HGNC Symbol;Acc:HGNC:23089]
	Digestibility	PITPNM3	PITPNM family member 3 [Source:HGNC Symbol;Acc:HGNC:21043]
Immune system activation	BT20	TNFRSF13B	Gallus gallus tumor necrosis factor receptor superfamily, member 13B (TNFRSF13B), mRNA. [Source:RefSeq mRNA;Acc:NM_001097537]
	BT22	DDT	Gallus gallus D-dopachrome tautomerase (DDT), mRNA. [Source:RefSeq mRNA;Acc:NM_001030667]
	BT22	CABIN1	Calcineurin binding protein 1 [Source:HGNC Symbol;Acc:HGNC:24187]
	BT28	MIF	Macrophage migration inhibitory factor [Source:UniProtKB/Swiss-Prot;Acc:Q02960]
	Breast yield	SOCS2	Suppressor of cytokine signaling 2 [Source:RefSeq peptide;Acc:NP_989871]
Cell signaling	BW21	EXOC6	Exocyst complex component 6 [Source:RefSeq peptide;Acc:NP_001012923]
	BW28	PPP1R3C	Protein phosphatase 1, regulatory subunit 3C [Source:HGNC Symbol;Acc:HGNC:9293]
	BT20	COPS3	Gallus gallus COP9 constitutive photomorphogenic homolog subunit 3 (Arabidopsis) (COPS3), mRNA. [Source:RefSeq mRNA;Acc:NM_001006163]
	BT20	NT5 M	5′,3′-nucleotidase, mitochondrial [Source:HGNC Symbol;Acc:HGNC:15769]
	BT20	RASD1	Gallus gallus RAS, dexamethasone-induced 1 (RASD1), mRNA. [Source:RefSeq mRNA;Acc:NM_001044636]
	BT22	CRKL	v-crk avian sarcoma virus CT10 oncogene homolog-like [Source:HGNC Symbol;Acc:HGNC:2363]
	BT28-20	MAP3K3	Mitogen-activated protein kinase kinase kinase 3 [Source:HGNC Symbol;Acc:HGNC:6855]
	BT28-20	DCAF7	Gallus gallus DDB1 and CUL4 associated factor 7 (DCAF7), mRNA. [Source:RefSeq mRNA;Acc:NM_001079504]
Apoptosis	Digestibility	XAF1	XIAP associated factor 1 [Source:HGNC Symbol;Acc:HGNC:30932]
	Breast yield	CRADD	Gallus gallus CASP2 and RIPK1 domain containing adaptor with death domain (CRADD), mRNA. [Source:RefSeq mRNA;Acc:NM_001030748]
Glucose	BW21	IDE	Insulin-degrading enzyme [Source:HGNC Symbol;Acc:HGNC:5381]
	BW28	TNKS2	Gallus gallus tankyrase, TRF1-interacting ankyrin-related ADP-ribose polymerase 2 (TNKS2), mRNA. [Source:RefSeq mRNA;Acc:NM_204341]
Disruption of cytoskeleton	BW21	KIF11	Gallus gallus kinesin family member 11 (KIF11), mRNA. [Source:RefSeq mRNA;Acc:NM_001031230]
Free radical damage	BT22	GSTT1	Gallus gallus glutathione S-transferase theta 1 (GSTT1), mRNA. [Source:RefSeq mRNA;Acc:NM_205365]
	Digestibility	TXNDC17	Thioredoxin domain containing 17 [Source:HGNC Symbol;Acc:HGNC:28218]
Blood vessel development	BT28-20	ACE	Gallus gallus angiotensin I converting enzyme (peptidyl-dipeptidase A) 1 (ACE), mRNA. [Source:RefSeq mRNA;Acc:NM_001167732]

All characterized genes within a 200-kb region, i.e. 100 kb upstream and 100 kb downstream of the SNP which was most frequently included in the MCMC iterations (post-burn-in), and is in the window explaining the largest amount of genetic variation for each trait

Body temperature (BT) measured on days 20, 22, 28, and the differential 28-20; body weight (BW) measured on days 21, 28, and the differential 28-21; digestibility, measured from ileal content, on day 28; percent of breast weight, calculated from percent of total body weight, and measured on day 28

## Discussion

The aims of this study were to identify and estimate the effect of QTL, and to identify positional candidate genes, for BT, BW, dry matter digestibility, and breast yield using a novel AIL under heat stress and a 600 K SNP panel for genotyping.

### Population used

Previous generations of this AIL were used for several QTL mapping studies and allowed the identification of many QTL including 257 QTL for growth and body composition [21–25], 93 for skeletal integrity [26], 51 for metabolic traits [27], and 12 for response to *Salmonella enteritidis* challenge [28–30]. Therefore, collectively, a wide range of traits has been associated with a large number of QTL in previous generations of this AIL. The continued erosion of LD in this population over subsequent generations, combined with the availability of more dense SNP panels, creates a unique opportunity to more finely map the location of QTL that are in LD with a causal mutation.

### Phenotypes and heritabilities

Phenotypic measurements for BT and BW consisted of repeated measures on individual birds. This allowed us to use both absolute measures and the differences between measures carried out before (pre-heat) and during heat treatments. Since measurements of dry matter digestibility and breast yield required euthanization, they were only performed after 7 days of heat treatment, on day 28.

A previous study reported a significant correlation between BT and survival in chickens during heat stress [31], which suggested that selection for BT during heat stress has potential to reduce mortality. In our study, heritabilities were low (0.03–0.11) for BT and higher (0.10–0.11) for absolute BT than that previously estimated for a broiler line i.e. 0.05 [32]. Heritabilities for changes in BT from pre-heat to acute and chronic heat conditions were both low i.e. 0.03, which could be due to the low precision and large variation of the measurements. This indicates that it will be challenging to genetically select for resistance to BT change during heat stress. More precise methods of BT measurement (e.g., infrared thermography) should be explored.

Heritabilities for BW were low to moderate (0.15–0.35), which agree with the heritabilities of 0.4 to 0.6 previously reported for BW in broiler lines [33]. Heritability for breast yield was moderate i.e. 0.15 and agreed with previously reported estimates [34].

In this study, dry matter digestibility was measured using a titanium oxide marker to calculate dry matter in the feed and ileal contents. We estimated a heritability of 0.33 for digestibility, which is similar to that (0.33 to 0.47) reported for broilers that were fed a wheat-based diet [35]. Many previous studies on feed conversion ratio in chickens reported moderate heritabilities [36–38]. Feed intake and conversion ratios, which are associated with digestibility, are arguably the most costly impacts of heat stress. Because the heritability estimated for digestibility during heat stress is moderate, there may be potential for improvement of this trait via selection.

Generally, heritabilities estimated for most traits in our study are lower than those previously reported, which is likely due to lower genetic variation within the population studied.

### Genome-wide association study

To date, seven QTL for BT have been reported on GGA2, 3, 4, 5, 6 and 11 [39–41], but none overlapped with those detected here. This absence of QTL overlap may be due to differences in experimental protocols since, in these previous studies [39–41], the traits that were measured were response to disease challenge and resting BT between lines selected for growth or fat accretion. In addition, these QTL may be population-specific.

The two QTL which co-localized for BT20 and BT28-20 and for BT20 and BT28 were near QTL for hematocrit on GGA14 [42] and GGA15 [39], respectively. One mechanism to regulate BT during periods of heat is to increase blood flow towards the body surface [43], and it has also been well documented that panting behaviour occurs under high temperatures [44]; both mechanisms result in changes in the blood system. Moreover, the co-localized QTL for BT on GGA15 was located near a QTL for corticosterone that was measured in response to manual restraint [45]. This co-localization suggests that it may be a good candidate for further investigation of the pleiotropic response to stress.

Heat stress specific QTL were identified for BT28 and BT28-20 on GGA26 and 27, respectively. Both regions present considerable overlap with previously reported QTL for growth [23, 24, 40, 46–49]. The large overlap between BT and QTL for growth is not surprising given the highly negative correlation between BT and growth during heat stress in chickens [50], although we attempted to account for this relationship by fitting BW as a co-variate in the GWAS analysis.

The co-localized QTL for all BW measurements on GGA6 was near a previously reported QTL for growth in many different chicken populations, including a broiler × layer cross [48, 49, 51, 52], White Plymouth Rock, New Hampshire and White Leghorn chickens [53], a commercial broiler line [54], high and low growth broiler lines [40], white leghorn x red jungle fowl [55], and the F2 broiler × Fayoumi generation used in the current study [23]. The QTL that we detected on GGA6 is confirmed by previously reported QTL in this region in a wide range of chicken populations, which suggests a conserved QTL, and supports our results. This region explained a relatively large percentage of the genetic variation for BW21 (3.0 %), BW28 (8.1 %), and BW28-21 (4.6 %), which confirms the importance of this QTL.

The co-localized QTL for BW21 and BW28 on GGA1 were also localized near QTL for growth that were previously reported in a large range of chicken populations including crosses between Silkie fowl and Cornish broiler [46], White Recessive Rock and Xinghua chicken [56], broiler and layer [49], and Shamo and White Plymouth Rock [57]. As observed for the co-localized QTL on GGA6, this region on GGA1 is probably highly conserved given the variety of populations for which QTL have been reported near this region.

Several QTL for average daily gain are located near the region where QTL for BW28 and BW28-21 co-localize on GGA4, in two chicken populations including a Silky fowl × White Plymouth rock cross [58], and a broiler × layer cross [49]. Again, this overlap between the QTL detected in our study and previously reported QTL supports our results.

Although it is interesting to discuss overlapping QTL for measurements performed at different phases, it is also relevant to examine the QTL that were identified specifically for traits measured during heat stress i.e. BW28 on GGA26 and BW28-21 on GGA10 and 21. Indeed, the QTL for BW28 on GGA26 was located within the 2-Mb QTL region for BT28 and as discussed above, many QTL for growth have been identified in this region. Similarly, QTL for growth have also been reported in the QTL regions on GGA10 and GGA21 that we detected here.

Although feed represents a large proportion of poultry production costs (51.8 % in 2001 and 68.7 % in 2008) and these costs continue to increase due to the increased demands for grain in other industries such as ethanol for fuel [59], very few QTL related to feed use have been identified (37 of the 4795 QTL listed in http://www.animalgenome.org). In a recent study that evaluated layer hens for feed intake and feed use and performed a GWAS using the 600 K array, eight QTL were identified [60], but none co-localized with the QTL reported here. The three QTL identified for digestibility were all located near previously reported QTL for growth-related traits. Very recently, Mignon-Grasteau et al. [61] identified a QTL for dry excreta weight on GGA19 within the same interval as that reported here for digestibility. The fact that the QTL regions for these two feed-use traits overlap provides evidence that this region on GGA19 is indeed a true QTL and should be further investigated given the economic importance of these traits to the poultry industry.

A strong QTL for breast yield was identified on GGA1. This region contains a large number of QTL related to abdominal fat and growth traits that were detected across diverse chicken populations (http://www.animalgenome.org). Surprisingly, no QTL for breast muscle has been reported in this region even in studies on previous generations of the same AIL. Thus, we suggest that this QTL may be specific to breast muscle growth during heat stress conditions. Furthermore, the QTL for breast yield that we detected on GGA15 and 22 overlap with previously reported QTL for breast muscle on GGA15 [40] and GGA22 [46]. The region on GGA1 warrants further investigation as a QTL specific to heat stress.

### Candidate genes

All positional candidate genes were identified for each trait within 200 kb of the most significant SNP. Cellular response to heat stress has been extensively reviewed and involves a range of biological mechanisms, i.e. inhibition of DNA synthesis, transcription, translation, cell cycle arrest, denaturation of proteins, enhanced degradation of proteins by ubiquitin and lysosomal pathways, disruption of the integrity of the cytoskeleton [62] and increased apoptosis [63]. In addition, heat stress induces metabolic changes and increased intracellular ion concentrations. Previously, Coble et al. [64] observed that, in broiler chickens, heat stress induced transcriptional changes and Morimoto [65] reported an increased expression of heat shock protein genes in response to heat stress. Heat shock proteins form an evolutionarily conserved family across all multicellular organisms [66].

Genes involved in the disruption of DNA synthesis, transcription, RNA processing, and translation were identified near the QTL for all traits analyzed in this study except for BW28 and BW28-21. We identified three genes that code for separate subunits of the mediator complex (MED 9, 15, and 31). The mediator complex is required for the regulation of eukaryotic RNA polymerase II transcripts [67]. In yeast, the mediator complex interacts directly with heat shock proteins and serves as a bridge between heat stress and transcriptional regulation of heat shock related genes [68].

Disruption of progression through the cell cycle and resulting apoptosis occur during cellular stress. We identified five genes that are involved in cell cycle progression that were located near some of the QTL detected in our study for all BT measurements and digestibility. Among the cell cycle checkpoints, two crucial checkpoints, between G1/S and G2/M transitions, are arrested in response to heat stress [69]. Apoptosis is induced during extreme stress conditions. In this study, we identified genes involved in apoptosis near QTL for both digestibility and breast yield.

Other categories of functions that were associated with the candidate genes detected in our study include glucose regulation, disruption of the cytoskeleton, free radical damage, and blood vessel development. The *ACE* gene, involved in blood vessel development, was located near the QTL for BT28-20, thus we hypothesize that it may play a role in reducing BT during periods of heat stress by enhancing blood flow to the body surface [43].

Genes involved in membrane permeability and changes in cellular ion concentrations were located near the QTL for BT, BW, and digestibility. In chickens, Ait-Boulahsen et al. [70] showed that Na^+^, K^+^, and Cl^−^ plasma levels increase in response to heat stress, which can have an effect on the endocrine system, for example as a secondary messenger, and subsequently on stress response. Two genes of this functional category were identified near the QTL for digestibility i.e. *SLC13A5* that encodes a citrate transporter and *PITPNM3* that encodes a calcium ion binding protein; both these genes are involved in ion movement, which is impacted by heat stress.

Genes related to the immune system were identified near the QTL for BT20 and BT28, and breast yield. It has been shown that, compared to animals not exposed to high ambient temperatures, laying hens that are exposed to cyclic heat stress have decreased T-cell and B-cell proliferations and antibody titre to sheep red blood cells and an increased total white blood cell count [71], which supports the hypothesis that the immune function is disrupted during heat stress. We identified the *MIF* and *DDT* genes near the QTL for BT22 and BT28, respectively. These genes function as proinflammatory cytokines involved in the immune response [72]. One of the hallmarks of inflammation is to increase BT. If inflammation can be suppressed in birds subjected to heat stress, this might decrease the negative impact of high ambient temperatures. The *SOCS2* gene that encodes a suppressor of cytokine signalling was identified near the QTL for breast yield and may be a good candidate gene for future studies on the mechanisms that influence breast muscle yield in chickens.

Candidate genes with a role in cell signalling were identified near the QTL for BT and BW. Cell signalling increases during response to stress. One gene of particular interest in cell signalling is the *MAP3K3* gene that was found near the QTL for BT28-20 since that the MAPK signalling pathway is known to be involved in the cellular response to stress [73].

Breast muscle yield is an extremely important trait because of its economic impact in the broiler industry. The 1-Mb window with the largest effect that we identified here was for breast yield and explained 15.4 % of the total genetic variation. The favourable allele of the SNP with the largest effect within the window that explained the largest proportion of genetic variation was fixed in the Fayoumi line but was also segregating in the broiler line; thus, it was not possible to determine which line contributed the favourable allele. The best candidate gene in this region is *SOCS2*, which has a role in suppressing cytokine signalling. We hypothesize that the effect of this QTL on muscle accretion may be heat-specific because no other QTL for breast yield was identified in this region.

## Conclusions

SNPs were identified for BT, BW, digestibility, and breast yield in a unique chicken AIL measured under heat stress. A major QTL for breast yield under heat stress explained more than 24 % of the genetic variation. Exploiting this information for genomic selection to breed heat-tolerant chickens is feasible. The QTL regions that we identified contain many genes with functions that suggest a role in response to heat stress and, thus, these genes are both positional and functional candidates.
